# Whole-Genome Sequencing Analysis of Methicillin-Resistant *Staphylococcus simulans* Causing Surgical Site Infection

**DOI:** 10.1128/genomeA.00555-16

**Published:** 2016-06-16

**Authors:** Jian Chen, Qiang Fang

**Affiliations:** Intensive Care Unit, The First Affiliated Hospital, College of Medicine, Zhejiang University, Hangzhou, China

## Abstract

*Staphylococcus simulans* is a normal part of the microbiota in humans and animals and is rarely associated with human invasive infections. We present here the genome sequence of *S. simulans* CJ16, which caused the first case of surgical site infection. Adhesion proteins, including fibronectin-binding protein (FnbA), elastin-binding protein (EbpS), and cell wall-anchored protein (SasA, SasF, and SasH), were detected in the genome, which might promote the survival of *S. simulans* on human skin and pathogenesis of infections.

## GENOME ANNOUNCEMENT

Surgical site infections, such as abscesses, wounds, and necrotizing fasciitis, were usually associated with contaminated surgical sutures. Most of these infections were due to pathogens capable of producing a biofilm, allowing microbial persistence against antimicrobial therapy (1). To date, *Staphylococcus aureus* is the most common pathogen isolated from surgical site infections, followed by coagulase-negative staphylococci (CoNS) (2). *Staphylococcus simulans* is a member of the CoNS and has been implicated as an opportunistic pathogen in humans and animals; it is usually recovered from cattle, sheep, and even hedgehogs (3–5). Previous studies have sequenced the whole genome of the *S. simulans* strain causing bovine mastitis (6). However, little is known about genetic determinants that contribute to virulence and survival. Here, we report the genome sequence of strain CJ16, which caused the first case of surgical site infection by *S. simulans*.

Genomic DNA was prepared as described previously (7). Libraries were constructed for sequencing with Nextera DNA kits (Illumina) and sequenced on the Illumina HiSeq 2000 system, according to standard Illumina protocols. The raw reads were trimmed and assembled as previously described (8). Predicted genes were identified using Glimmer (9). tRNAscan-SE (10) was used to find tRNA genes, whereas ribosomal RNAs were found by using RNAmmer (11). The draft genome was annotated by Rapid Annotations using Subsystems Technology (RAST) (12). Coding sequences were analyzed to detect toxin genes by using VirulenceFinder (http://cge.cbs.dtu.dk/services/VirulenceFinder/). Putative phage sequences were identified by PHAST (13). CRISPRFinder was used to screen for the presence of clustered regularly interspaced short palindromic repeat (CRISPR) arrays (14).

The assembly genome of *S. simulans* CJ16 comprises an approximately 2.67-Mbp chromosome. The sequence consists of 112 contigs and 2,517 coding sequences. It does not carry any integrated or replicating plasmids.

The genome of CJ16 contains several genes that might be of relevance to skin survival, in keeping with the identity of *S. simulans* as an opportunistic pathogen. Predicted adhesion proteins involved in colonization and host protein binding, such as autolysin, fibronectin-binding protein (*fnbA*), elastin-binding protein (*ebpS*) (15), cell wall-anchored protein (*sasA*, *sasF*, and *sasH*) (16), and several additional genes encoding host protein-binding motifs were identified in the genome. Adhesion-encoding genes have been commonly identified in *S. aureus* isolates, and they have been recognized as being more associated with invasive infections (15).

The *isd* locus was found to encode factors that bind human hemoproteins, remove the heme molecule, and transport heme through the cell wall during infection (17). To date, the *isd* locus only has been detected in *S. aureus* and *Staphylococcus lugdunensis* (18). Here, we identified an *isd* locus from the genome of *S. simulans* CJ16. The overall gene organization of the *S. simulans* Isd locus and *S. aureus* Isd locus is very similar. The major difference between *S. simulans* and *S. aureus* Isd is the absence of the IsdI heme oxygenase from *S. simulans*. These data may indicate that CJ16 is able to cause severe infections.

The availability of the *S. simulans* CJ16 genomic sequence data will contribute to easier genetic manipulation of this strain and will enhance further studies in the future.

### Nucleotide sequence accession numbers.

The whole-genome shotgun project of *S. simulans* CJ16 has been deposited at DDBJ/EMBL/GenBank under the accession no. LJSL00000000. The version described in this paper is version LJSL01000000.
